# Gender and racial differences in first and senior authorship of high-impact critical care randomized controlled trial studies from 2000 to 2022

**DOI:** 10.1186/s13613-023-01157-2

**Published:** 2023-06-27

**Authors:** Subhash Chander, Sindhu Luhana, Fnu Sadarat, Lorenzo Leys, Om Parkash, Roopa Kumari

**Affiliations:** 1 Department of Internal Medicine, Mount Sinai Beth Israel, 281 1st Ave, New York, NY 10003 USA; 2 Department of Medicine, AGA Khan University, Karachi, Pakistan; 3 Department of Medicine, University at Buffalo, New York, USA; 4 Department of Pulmonary and Critical Care, Mount Sinai West and Morningside, New York, USA; 5 Department of Medicine, Albert Einstein, Montefiore Medical Centre, New York, USA; 6 Department of Pathology, Mount Sinai Morningside and West, New York, USA

**Keywords:** Authorship, Critical care, Gender disparities, Racial disparities, Ethnic disparities, RTCs

## Abstract

**Background:**

Females and ethnic minorities are underrepresented in the first and senior authorships positions of academic publications. This stems from various structural and systemic inequalities and discrimination in the journal peer-review process, as well as educational, institutional, and organizational cultures.

**Methods:**

A retrospective bibliometric study design was used to investigate the representation of gender and racial/ethnic groups in the authorship of critical care randomized controlled trials in 12 high-impact journals from 2000 to 2022.

**Results:**

In the 1398 randomized controlled trials included in this study, only 24.61% of the first authors and 16.6% of the senior authors were female. Although female authorship increased during the study period, authorship was significantly higher for males throughout (Chi-square for trend, *p* < 0.0001). The educational attainment [*χ*^2^(4) = 99.2, *p* < 0.0001] and the country of the author's affiliated institution [*χ*^2^(42) = 70.3, *p* = 0.0029] were significantly associated with gender. Male authorship was significantly more prevalent in 10 out of 12 journals analyzed in this study [*χ*^2^(11) = 110.1, *p* < 0.0001]. The most common race/ethnic group in our study population was White (85.1% women, 85.4% males), followed by Asians (14.3% females, 14.3% males). Although there was a significant increase in the number of non-White authors between 2000 and 2022 [*χ*^2^(22) = 77.3, *p* < 0.0001], the trend was driven by an increase in non-White male and not non-White female authors. Race/ethnicity was significantly associated with the country of the author’s affiliated institution [*χ*^2^(41) = 1107, *p* < 0.0001] but not with gender or educational attainment.

**Conclusions:**

Persistent gender and racial disparities in high-impact medical and critical care journals underscore the need to revise policies and strategies to encourage greater diversity in critical care research.

## Background

Gender and racial diversity in authorship have been associated with increased productivity in the workforce as well as increased citations. In addition, social identities can lead to medical advancements in areas greatly neglected, suggesting a myriad of scientific and social benefits in increasing diversity in scientific research [1–3].

While several studies have shown the underrepresentation of women physician-scientists in the authorship of research articles [4–7], the problem may be particularly exacerbated in critical care. For example, women comprised less than 40% of single, first, or senior authors of critical care literature published in 2016, and critical care was the only medical discipline with a negative annual rate of change (− 1% per year) in women authorship [6]. Similarly, racial/ethnic disparities in medical research output have been widely reported [7–12]. Asians, Hispanics, and Blacks are severely underrepresented in first senior authorship positions than non-Hispanic White in biomedical literature [13] and even more so in highly prestigious journals such as the Journal of the American Medical Association (JAMA) or the New England Journal of Medicine (NEJM) [7]. However, racial/ethnic disparities in medical research output are not as widely studied as gender disparities, with a lack of studies on racial/ethnic disparities in the critical care subspecialty.

Nonetheless, it is likely that gender and racial/ethnic disparities both exist in critical care literature and may have been aggravated by the COVID-19 pandemic. For instance, Madsen et al. [14] demonstrated that women had 17–24% lower publication output than men during 2019–2020 versus 2017–2019. Moreover, the widening gender gap was more pronounced in early- to mid-career versus senior-career women scientists [14]. Women in clinical medicine fared poorly in publication output compared to those in basic medicine, biology, or chemistry during the pandemic [14]. Similarly, Naidoo et al. [15] showed that only 3.9% of COVID-19-related articles in top-tier medical journals were pertinent to Africa, 36.2% with an African first author, 19.1% with an African last author, and 13.8% with both African first and last author.

Given these gaps in the literature, this study aimed to evaluate the degree of gender and racial disparities in first and senior authorship of randomized controlled trials (RCTs) in critical care through bibliometric analysis of seven specialized and five generic medical journals from 2000 to 2022. We hypothesized that gender and racial disparities would persist throughout these 22 years, with white male authors dominating first and senior authorship. In addition, country, educational attainment, and journal type were also explored in their relationship with gender and racial inequalities in authorship.

## Methods

### Study design

A retrospective bibliometric study design was used for this study, as reported in prior studies [16–18], to ensure comparability with the existing literature in this domain. Twelve journals were selected based on their impact factor according to the Journal Citation Reports and comprised seven journals specializing in critical care and five large general medicine. The specialization of the selected journals was ascertained from the scope, aims, and objectives available from the journal website. The seven specialized journals include the American Journal of Critical Care (AJCC; 2021 IF = 2.207), the American Journal of Respiratory and Critical Care Medicine (AJRCCM; 2021 IF = 30.528), Critical Care (2021 IF = 19.334), Critical Care Medicine (CCM; 2021 IF = 9.296), Intensive Care Medicine (ICM; 2021 IF = 41.787), the Journal of Critical Care (JCC; 2021 IF = 4.298), and the Journal of Intensive Care Medicine (JICM; 2021 IF = 2.889). The five general medical journals include British Medical Journal (BMJ; 2021 IF = 93.33), Chest (2021 IF = 10.262), JAMA (2021 IF = 157.335), Lancet (2021 IF = 202.731), and NEJM (2021 IF = 176.079).

### Search strategy

The literature search was conducted using the litsearchr and easyPubMed packages for R, version 4.3.0 [19], using a combination of keywords and MeSH terms such as "Gender Differences" OR "Sex Factors") AND Authorship AND ("Critical Care" OR "Intensive Care Units") AND ("Randomized Controlled Trials as Topic" OR "Clinical Trials as Topic") AND ("Ethnic Groups" OR "Race Factors") AND ("Publication Bias" OR "Journal Impact Factor") AND ("Research Personnel" OR "Leadership") AND ("Healthcare Disparities" OR "Social Justice") AND ("Research Design" OR "Epidemiologic Research Design") AND ("2000/01/01"[Date—Publication]: "2022/12/31"[Date—Publication]).

### Inclusion and exclusion criteria

Critical care RCTs published between January 2000 and December 2022 in the selected high-impact journals were eligible for inclusion. We excluded articles published in a non-English language, brief communications, commentaries, review articles, non-randomized trials, case reports, meta-analyses, and studies with insufficient authorship information.

### Data extraction

Two independent researchers (S.C and R.K) reviewed articles for eligibility and extracted data using a standardized data extraction form. Any discrepancies were resolved through discussion or by consulting a third researcher. The extracted information included publication year, journal name, impact factor, first author name and affiliation, senior author name and affiliation, author gender, and author race/ethnicity.

### Gender and racial/ethnic classification

We determined the gender of the first and senior authors based on their names, online biographies, or publicly available photographs when necessary. The racial and ethnic background of the authors was inferred using a combination of their names, affiliations, and online biographies. We categorized the authors into five main racial/ethnic groups: White, Asian, Black, Hispanic/Latino, and Arab.

The data of first and senior authors from studies meeting the inclusion criteria were combined for analysis.

### Statistical analysis

Categorical variables were described in frequencies and percentages. Demographic characteristics and journal variables were compared using the Chi-square test. All analyses were performed using R software, version 4.1.3. A *p-*value of < 0.05 was considered statistically significant.

## Results

**Fig. 1:**
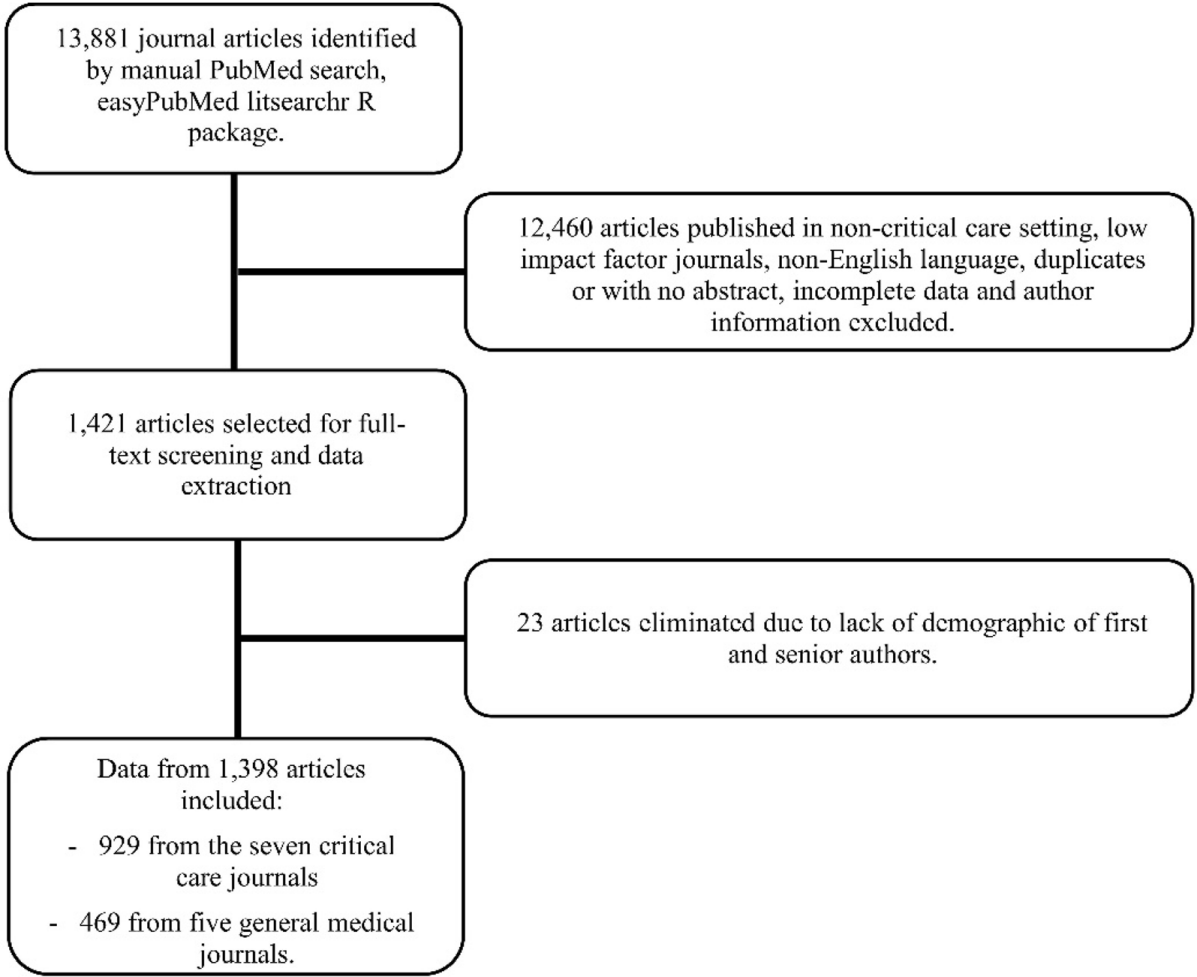
Flowchart of inclusion/exclusion process of journal articles

A total of 13,881 RCTs published between 2000 and 2022 in 12 journals were initially reviewed, of which 12,460 did not meet the inclusion criteria. A Google search was performed on the remaining 1421 RCTs to ascertain the first and senior authors’ gender, race, country, and educational attainment. Subsequently, 23 articles were excluded as the demographic characteristics of the first and senior authors could not be ascertained. Finally, 929 articles from journals specializing in critical care research and 469 articles from general medical journals were included in this study. The flow of the study selection process is described in Fig. 1.

### Trends in female authorship

**Fig. 2:**
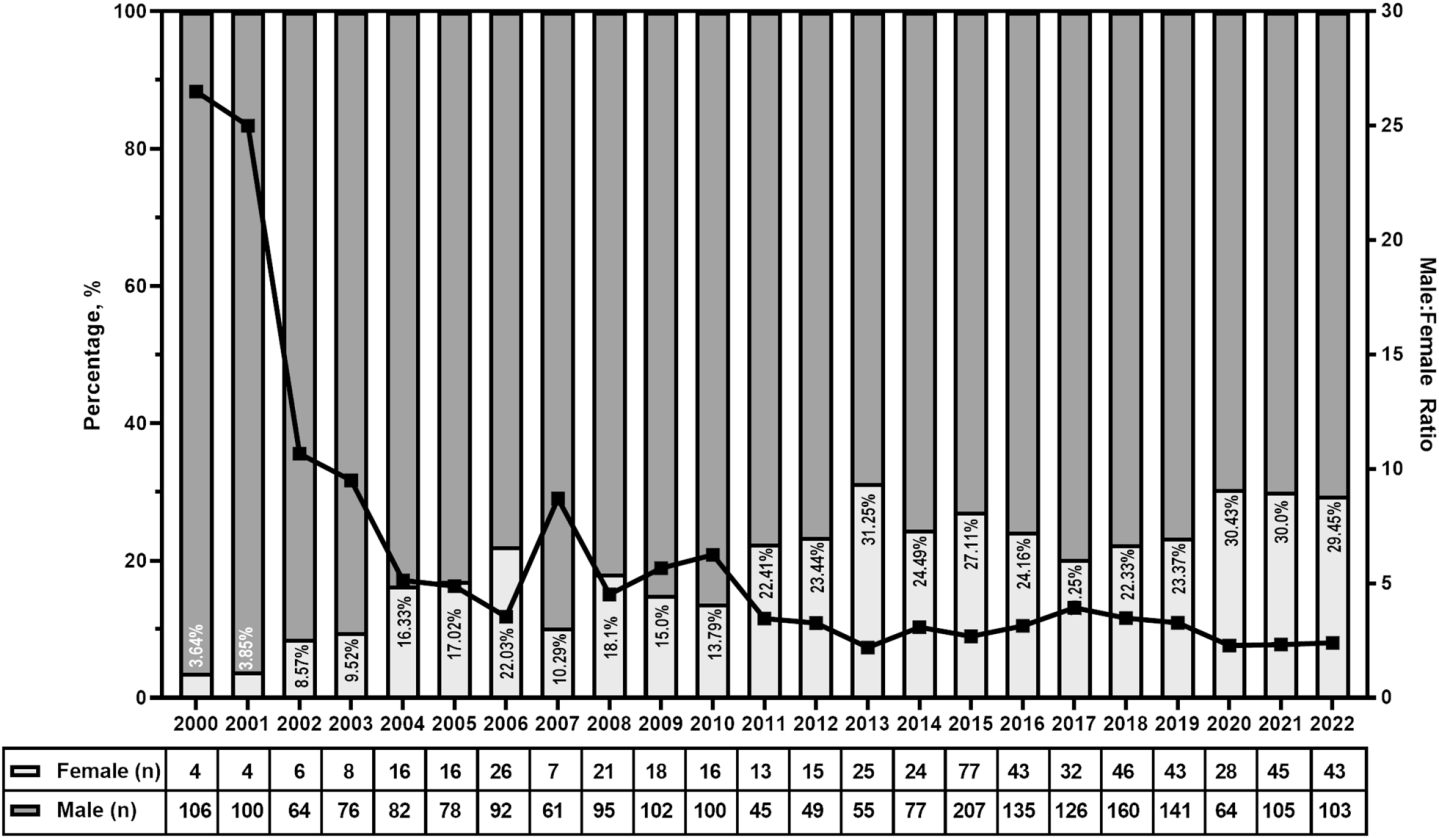
Annual proportions (bar) and ratios (line) of male and female authors. Chi-square for trend *p* < 0.0001

The 1398 RCTs in this study provided a pool of 2796 authors in first and senior authorship positions. There was a significantly greater proportion of males in both the first (24.6% vs. 75.4%) and senior (16.6% vs. 83.4%) authorship positions (Table 1). The proportion of total female authors in the combined pool of first and senior authors gradually increased from 2000 to 2013, when the number peaked (Fig. 2). Female authorship remained relatively steady from 2014 to 2019, increasing to around 30% in the post-COVID-19 pandemic years of 2020, 2021, and 2022 (Fig. 2). However, authorship was significantly higher for males throughout the study period (Fig. 2; Chi-square for trend, *p* < 0.0001).

**Table 1:** Demographic characters of authors

Variables	Female *n* (%)	Male *n* (%)	*p*
Authorship position			
First author	344 (24.6)	1054 (75.4)	< 0.0001
Senior author	232 (16.6)	1166 (83.4)	
Race/ethnicity			
White	490 (85.1)	1895 (85.4)	0.9
Black	3 (0.5)	8 (0.4)	
Asian	74 (12.8)	276 (12.4)	
Hispanic	6 (1.0)	28 (1.3)	
Arab	3 (0.5)	13 (0.6)	
Education attainment			
MD	408 (70.8)	1672 (75.3)	< 0.0001
MD, PhD	51 (8.8)	384 (17.3)	
PhD	41 (7.1)	52 (2.3)	
Masters’	61 (10.6)	86 (3.9)	
Others/unknown	15 (2.6)	26 (1.2)

Our study sample comprised authors affiliated with institutions in 42 countries (Table 2). The country of the author's affiliated institution was significantly associated with gender [Table 2; *χ*^2^(42) = 70.3, *p* = 0.0029]. Over 85% of the female authors and 83% of male authors came from just 10 out of 42 countries represented in this study, with most female authors from the USA (36.6%) followed by Canada (10.9%), the UK (7.99%), France (6.6%), Australia (6.1%), the Netherlands (5.4%), Belgium (5.2%), Germany (2.8%), Denmark (2.3%), and Spain (2.1%) (Table 2).

**Table 2:** Country of affiliated institution of female and male authors

Country of affiliated institution	Female *n* (%)	Male *n* (%)	*p*
USA	211 (36.6)	857 (38.6)	0.0029
Canada	63 (10.9)	165 (7.4)	
UK	46 (8.0)	158 (7.1)	
France	38 (6.6)	219 (9.9)	
Australia	35 (6.1)	119 (5.4)	
Netherlands	31 (5.4)	79 (3.6)	
Belgium	30 (5.2)	58 (2.6)	
Germany	16 (2. 8)	73 (3.3)	
Denmark	13 (2.3)	51 (2.3)	
Spain	12 (2.1)	64 (2.9)	
China	10 (1.7)	31 (1.4)	
Austria	9 (1. 6)	22 (1.0)	
Brazil	9 (1. 6)	22 (1.0)	
Greece	8 (1.4)	24 (1.1)	
Italy	6 (1.0)	76 (3.4)	
Switzerland	6 (1.0)	20 (0.9)	
Finland	5 (0.9)	11 (0.5)	
India	4 (0.7)	18 (0.8)	
Iran	4 (0.7)	18 (0.8)	
Japan	3 (0.5)	35 (1.9)	
Saudi Arabia	2 (0.3)	10 (0.4)	
Thailand	2 (0.3)	9 (0.4)	
Sweden	2 (0.3)	7 (0.3)	
Norway	2 (0.3)	2 (0.1)	
Korea	1 (0.2)	11 (0.5)	
Taiwan	1 (0.2)	9 (0.4)	
Turkey	1 (0.2)	9 (0.4)	
Colombia	1 (0.2)	3 (0.1)	
Singapore	1 (0.2)	2 (0.1)	
Egypt	1 (0.2)	1 (0.05)	
Malaysia	1 (0.2)	1 (0.05)	
Estonia	1 (0.2)	0 (0)	
Kenya	1 (0.2)	0 (0)	
New Zealand	0 (0)	9 (0.4)	
Ireland	0 (0)	6 (0.3)	
Scotland	0 (0)	6 (0.3)	
Czech Republic	0 (0)	4 (0.2)	
Israel	0 (0)	4 (0.2)	
Cameroon	0 (0)	2 (0.1)	
Chile	0 (0)	2 (0.1)	
South Africa	0 (0)	2 (0.1)	
Portugal	0 (0)	1 (0.05)	

In terms of educational attainment, a significantly greater proportion of males had MD (70.8% vs. 75.3%) and MD + Ph.D (8.8% vs. 17.3%) degrees, while greater proportions of female authors reported Ph.D. (7.1% vs. 2.3%) or Masters (10.6% vs. 3.9%) degrees [Table 1**;** *χ*^2^(4) = 99.2, *p* < 0.0001].

**Fig. 3:**
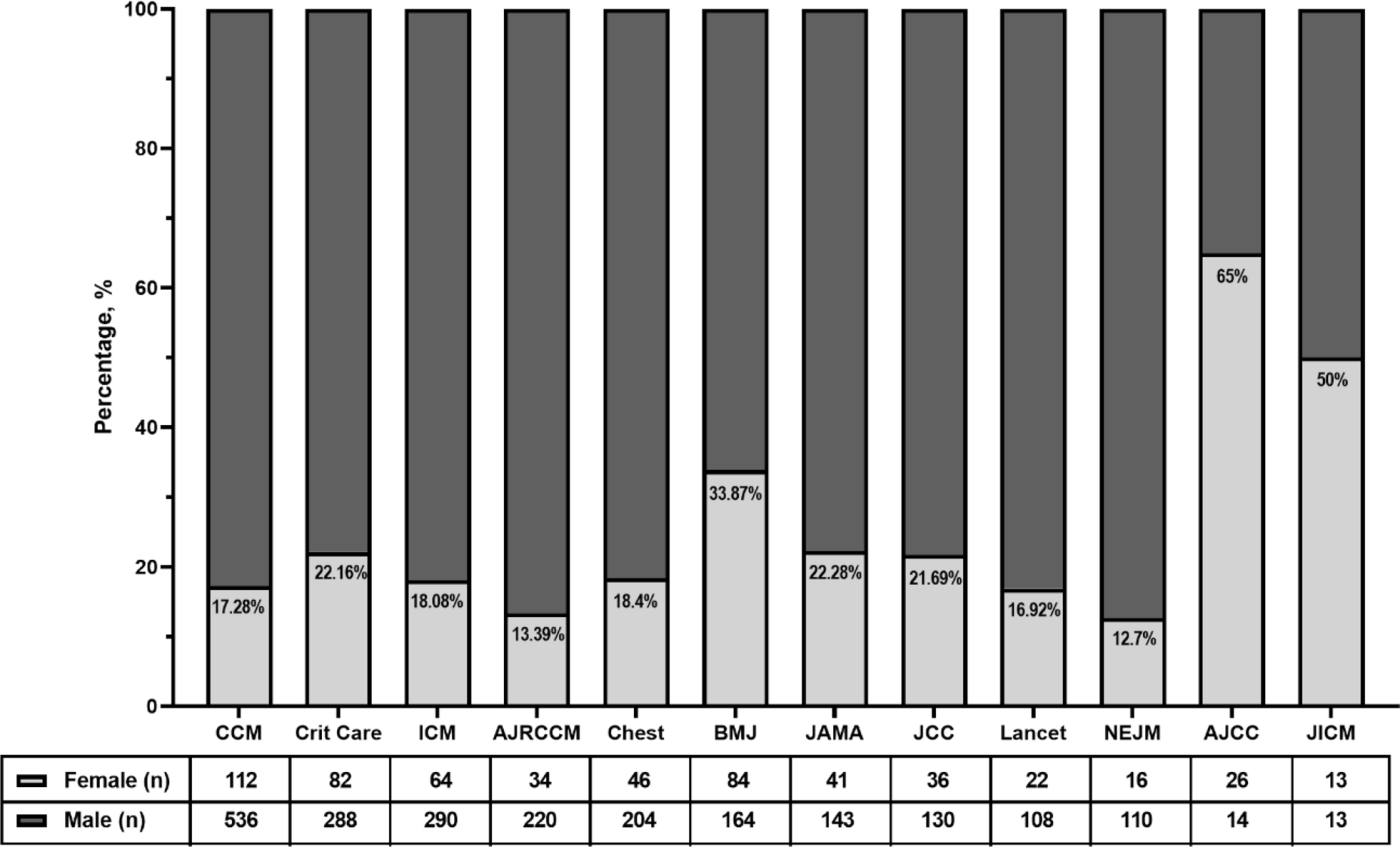
Gender of Authorship by Journal of Publication. *AJCC* American Journal of Critical Care, *AJRCCM* American Journal of Critical Care Medicine, *BMJ* British Medical Journal, *CCM* Critical Care Medicine, *ICM* Intensive Care Medicine, *JCC* Journal of Critical Care, *JICM* Journal of Intensive Care Medicine, *JAMA* Journal of American Medical Association, *NEJM* New England Journal of Medicine

AJCC had the highest proportion of female authorship during the study period (65%), followed by the JICM (50.0%) and BMJ (33.9%) (Fig. 3). Male authorship was significantly more prevalent than female authors in 10 out of 12 journals analyzed in this study [Fig. 3**;** *χ*^2^(11) = 110.1, *p* < 0.0001].

### The trend in race/ethnicity distribution in authorship

**Fig. 4:**
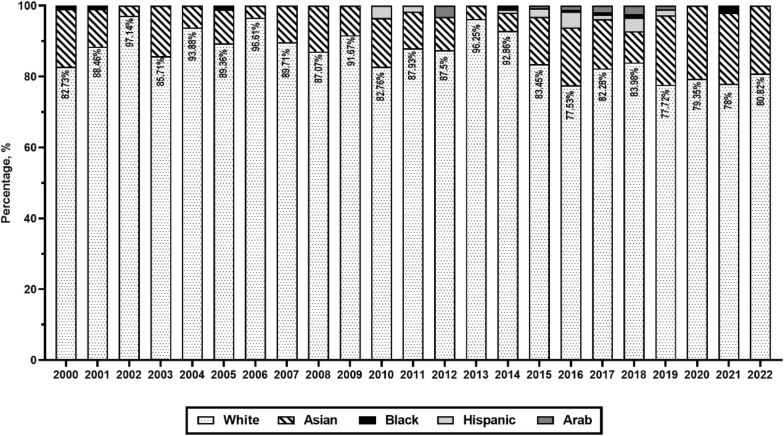
Annual proportions authors by race/ethnicity. Chi-square for trend *p* < 0.0001

The most common race/ethnic group in our study population was White (85.1% women, 85.4% males), followed by Asians (14.3% females, 14.3% males) (Table 1). Although race was not associated with gender (*p* = 0.9), there was a significant increase in the number of non-White authors between 2000 and 2022 [Fig. 4; *χ*^2^(22) = 77.3, *p* < 0.0001]. It is noteworthy that the increase in the number of non-White authors was driven by an increase in male [*χ*^2^(22) = 63.7, *p* < 0.0001] and not female [*χ*^2^(22) = 28.8, *p* = 0.1] authors (data not shown in tables).

Furthermore, there was no significant association between race and educational attainment [*χ*^2^(4) = 2.0, *p* = 0.73] with similar proportions of White and non-White authors reporting MD (74.3% vs. 74.9%), MD + Ph.D. (15.5% vs. 16.1%), Ph.D. (5.5% vs. 3.9%), Master's (1.4% vs. 1.7%) and other/unknown (3.3% vs. 3.4%) degrees. However, race/ethnicity was significantly associated with the country of the author's affiliated institution [*χ*^2^(41) = 1107, *p* < 0.0001], with two-thirds of non-White authors hailing from six countries: USA (28.9%), China (10.0%), Japan (8.5%), Canada (7.3%), the UK (6.8%), and India (5.3%).

## Discussion

This study provides the first evidence for the underrepresentation of women and non-White racial/ethnic groups in critical care RCTs over the past two decades. While female authorship has increased over time, they still feature as first or senior authors in only 30% of the critical care RCTs annually. Moreover, our analysis indicates regional disparities, with authors of both genders predominantly affiliated with institutions in the United States, Canada, and the UK. Similarly, although non-White authorship has increased over time, this trend was primarily driven by an increase in non-White males. Furthermore, there were no significant differences in first and senior authorship by race when gender was considered; female authors were predominantly White, and the combined proportion of Asian, Hispanic, and Black authors comprised less than 15% of female authors. These findings suggest that the underrepresentation of women and non-White racial/ethnic groups in critical care research are independent issues that require focused interventions.

Although there is a paucity of research exploring individual and systemic barriers that lead to the underrepresentation of racial/ethnic groups in medical research or publication output, the underrepresentation of women has been well studied. We identified four studies that have previously assessed gender disparity in authorship in critical care literature, albeit with some methodological differences. Nasrullah et al. [18] reported 29.1% and 21.2% females in first and senior authorship positions between 2019 to 2021. While our study period was much more extended, our study sample had a similar prevalence of female authorship between 2019 and 2021 (29.2% and 22.5%, respectively). Similarly, Vranas et al. [16] reported 30.8% and 19.5% of females as first and senior authors between 2008 and 2018, with a small but statistically significant increase in the annual rate of change for female authorship (0.44% for first and 0.51% for senior authorship) while the corresponding prevalence in our study sample during this period was and 26.9% and 17.7%, respectively. It is noteworthy that Nasrullah et al. [18] and Vranas et al. [16] did not restrict their analysis to RCTs. Given that our analysis is highly consistent with these earlier studies despite the methodological differences indicates that similar gender disparity may exist in the critical care literature irrespective of the study design.

In contrast, L Holman, D Stuart-Fox and CE Hauser [6] reported 39%, 27.3%, and 35.3% prevalence of females in first, last, and all authorship positions between 2002 and 2016, while Ravi et al. [20] reported a 35% prevalence in all authorship positions between 2016 and 2020. However, these studies were global studies that utilized all articles indexed in PubMed’s MEDLINE database during the study period. In comparison, Nasrullah et al. [18] included studies from the top 20 high-impact journals, Vranas et al. [16] included articles from 40 most frequently cited journals, and the current study sampled RCTs from 12 top-tier journals (7 specialized and five general medicine) that publish critical care-related articles. Therefore, it is plausible that more women in critical care research publish in lower-impact than high-impact journals. There is an indication of this in our study sample: women authors outpaced men in two of the 12 journals, which incidentally also had the lowest impact factors of the journals sampled in this study and contributed the lowest number of RCTs to our study. The study by Vranas et al. [16] provided more direct evidence by demonstrating that female first authors had 30% higher odds of publishing in lower-impact journals than male first authors.

There could be several reasons for the gender disparity in critical care literature. Despite the closing gender gap in medical schools [21–23], fewer women physicians move up the academic hierarchy [24, 25]. For instance, an analysis of membership data of the World Federation of Societies of Intensive and Critical Care Medicine showed that women were underrepresented in leadership positions in critical care organizations, critical care medicine boards and councils, and faculty representation at symposia despite an increase in women trainee and specialist [26]. In addition, low representation of women has been noted in the editorial board of critical care journals [18, 27] and as speakers in critical care conferences [28].

Original, peer-reviewed publications in high-impact journals are used as faculty performance indicators for promotions, grants, funding, and other resource allocations [17]. A growing body of evidence indicates that women researchers may have limited access to mentoring, networking, or funding opportunities, further compounded by higher work–life commitments and patriarchal organizational setup [29, 30]. For example, men spend more time in research, service, and administrative roles, while women have a higher teaching load, severely restricting their research workload [29, 31]. Even before the pandemic, females were involved in significantly fewer research leadership positions, led fewer funded research studies, and applied for fewer research grants than males [32]. Further, pay and position inequities are further exaggerated when women in academia are also mothers.

Moreover, first-time female authors tend to publish in low-impact journals, which may be another reason for their underrepresentation in high-impact journals [16, 33, 34]. However, female primary leadership in RCTs has been reduced to half over the last several years, suggesting an imbalance in access and funding [32].

In any case, the arrested career growth of women physician-researchers is perhaps the most crucial driver of the widening gender gap in critical care authorships. Recent studies indicate that the odds of female co-authorship are 1.9-fold higher when the senior author is female [16, 17]. With the dwindling number of women in leadership positions within the critical care workforce [26], further exacerbation of the gender gap in authorship is expected.

Statistics related to racial and ethnic minorities in critical care research are somewhat more mystifying, as tracking the race and ethnicity of authors is relatively new. Structural racism, including inequalities in grants and funding opportunities for research [35–37], is a systemic barrier to researchers from Black and other racial minorities. For instance, White applicants had a 19% funding rate for the R01 award from the National Institutes of Health, while Black participants had a funding rate of 11.8% [38]. Similarly, while White women researchers were as likely as White men to receive an R01 award from the National Institutes of Health, non-White women were less likely to receive funding than White women [35].

More recently, Ginther et al. [39] showed that Black researchers reported fewer publications in their R01 award application that were less frequently cited than those included by White researchers in their applications. This explained, at least in part, the Black/White funding gap, and the authors hypothesized that Black researchers might not receive the same research training and mentoring opportunities in their doctoral programs [39]. Indeed, another study by Osseo-Asare et al. [40] seems to support the notion that minority physicians have limited training and mentorship opportunities. In the current study, race was significantly associated with educational attainment; Arab, Asian, Hispanic, and White authors were more likely to have a medical degree, while Black authors were likelier to report others/unknown degree status.

Because our analysis was not limited to the United States and we sampled studies from all countries, our findings must also be considered in the context of countries with predominantly non-White populations, poorer gender representation in the research workforce, and with institutions considered less prestigious than in high-income countries. Scientists from prestigious organizations benefit the most from a fast-track peer-reviewed process, especially with single-blinded review procedures in place [33]. Moreover, some studies have documented global disparities in underrepresentation, with fewer journal submissions coming from low- and middle-income countries and the most underrepresentation of authorship occurring in low-income countries [33, 41].

### Limitations

This research has several limitations. While factors contributing to the underrepresentation of female and minority researchers in high-impact journal articles are a well-researched area, factors contributing to lower participation rates cannot be established in this research study. The data may not include all prospective studies published in the 12 journals selected between 2010 and 2021. However, employing software for article selection minimizes researcher bias in the process. Although a manual process was used to confirm the author's gender, there is room for human error, given that this study analyzed a pool of 2796 authors. Finally, by examining only males and females, this research excludes non-binary, trans, and non-conformity individuals.

## Conclusion

The findings of this study confirm previous empirical evidence that females and minorities have not yet reached equality in research compared to their White male counterparts. Demonstrating gender and racial disparities in high-impact critical care journals underscores the need for revised policies and strategies to encourage greater gender, racial, and ethnic diversity in peer-review processes and scholarly research fields. In addition to increased transparency, mitigation strategies could include review teams that are gender- and racially balanced. Increasing funding and grant opportunities in addition to educational, career, and mentoring experiences in those most underrepresented should be among the top institutional priorities to promote gender, racial, and ethnic equality, and justice.

## Data Availability

The datasets used and/or analyzed during the current study are available from the corresponding author upon reasonable request.

## Abbreviations

AJCC: American Journal of Critical Care
AJRCCM: American Journal of Critical Care Medicine
BMJ: British Medical Journal
CCM: Critical Care Medicine
ICM: Intensive Care Medicine
JCC: Journal of Critical Care
JICM: Journal of Intensive Care Medicine
JAMA: Journal of American Medical Association
NEJM: New England Journal of Medicine
ICU: Critical care unit
CI: Confidence interval
NIH: National Institutes of Health
